# An object-based sparse representation model for spatiotemporal image fusion

**DOI:** 10.1038/s41598-022-08728-6

**Published:** 2022-03-23

**Authors:** Afshin Asefpour Vakilian, Mohammad Reza Saradjian

**Affiliations:** School of Surveying and Geospatial Engineering, University College of Engineering, University of Tehran, Tehran, 1439957131 Iran

**Keywords:** Electrical and electronic engineering, Information technology

## Abstract

Many algorithms have been proposed for spatiotemporal image fusion on simulated data, yet only a few deal with spectral changes in real satellite images. An innovative spatiotemporal sparse representation (STSR) image fusion approach is introduced in this study to generate global dense high spatial and temporal resolution images from real satellite images. It aimed to minimize the data gap, especially when fine spatial resolution images are unavailable for a specific period. The proposed approach uses a set of real coarse- and fine-spatial resolution satellite images acquired simultaneously and another coarse image acquired at a different time to predict the corresponding unknown fine image. During the fusion process, pixels located between object classes with different spectral responses are more vulnerable to spectral distortion. Therefore, firstly, a rule-based fuzzy classification algorithm is used in STSR to classify input data and extract accurate edge candidates. Then, an object-based estimation of physical constraints and brightness shift between input data is utilized to construct the proposed sparse representation (SR) model that can deal with real input satellite images. Initial rules to adjust spatial covariance and equalize spectral response of object classes between input images are introduced as prior information to the model, followed by an optimization step to improve the STSR approach. The proposed method is applied to real fine Sentinel-2 and coarse Landsat-8 satellite data. The results showed that introducing objects in the fusion process improved spatial detail, especially over the edge candidates, and eliminated spectral distortion by preserving the spectral continuity of extracted objects. Experiments revealed the promising performance of the proposed object-based STSR image fusion approach based on its quantitative results, where it preserved almost 96.9% and 93.8% of the spectral detail over the smooth and urban areas, respectively.

## Introduction

Emerging remote sensing satellite instruments with various spatial and temporal resolutions have made spectacular improvements in the variety of available data^1^. However, it is yet impossible to achieve temporally dense global satellite images with high spatial resolution simultaneously due to the technological challenge or budget limitations^2^. Therefore, a trade-off between high spatial resolution and dense temporal coverage is always considered when designing a satellite sensor. Another limitation of achieving dense high-spatial-resolution optical images is the problem of gaps due to the cloud and snow cover in the captured scenes^3,4^. Lack of frequent data directly impacts the dynamic monitoring of high-frequency phenomena. Therefore, the high demand for fine spatial resolution satellite images with frequent temporal coverage has changed the focus of recent studies to fuse available temporally dense coarse spatial resolution images with high spatial resolution images^5^. Spatiotemporal satellite image fusion is a cost-effective solution to reduce the data gap resulting from cloud and snow cover and long revisit time intervals of a fine spatial resolution instrument^6^. Applications of dense, fine spatial resolution fused images might include high-frequency phenomena detection^7^, land use classification^8^, global-scale forest cover mapping^9^, monitoring earth hazards^10^, and dehazing^11–14^. However, color distortion is often a problem in the fusion methods due to the different environmental circumstances at the time of image acquisition^15^.

Many spatiotemporal models have been proposed for remote sensing applications; however, they were applied over simulated data, and a few were capable of dealing with changes (e.g., conversion of ground features and vegetation phenology changes) in real satellite scenes. In the case of simulated data, a coarse image is usually the downsampled version of an existing satellite image in which neighboring pixels are aggregated corresponding to the ground sample distance (GSD) of the target satellite image. More complex simulated data use pixel shifting and linear transformation to simulate geometric and radiometric errors, respectively. This indicates that simulated data are too preliminary to provide real experimental data. There are three main factors in real spatiotemporal image fusion applications that can affect the results that are misregistration of geometric features, radiometric inconsistency between fine and coarse input images, and spatial resolution ratio7. Lack of studies to deal with spatiotemporal fusion approach over real satellite data and evaluate the interpretability of the results and technical limitations towards accurate fused results is also a problem. This study aims at introducing, implementing, and evaluating a spatiotemporal fusion approach over real satellite images that can deal with the three mentioned factors.

A spatiotemporal fusion of the fine spatial resolution Sentinel-2 multispectral imager (MSI) data with coarse spatial resolution Landsat-8 optical land images (OLI) data in the visible and near-infrared (Vis/NIR) wavelengths with 10 m and 30 m GSD, respectively, is proposed in this study to produce more cloud-free fine MSI images. The results obtained from the proposed approach were carried out and compared to results obtained from well-known spatiotemporal fusion approaches in terms of spatial and spectral distortions using a quality assessment step.

### Main contributions

Compared with existing spatiotemporal fusion approaches that focus on simulated input data to avoid the effect of inevitable spectral distortions and spatial artifacts in real applications, this paper proposes a new object-based image fusion framework based on edge pixels in satellite data to deal with real spatiotemporal image fusion tasks. The proposed object-based approach is called spatiotemporal sparse representation (STSR). The main contributions of this study are summarized as follows:

1. It alleviates the main drawback of spatiotemporal fusion approaches that is the inconsistency of the predicted results in dealing with real satellite data.
2. The object-based procedure towards the spatiotemporal fusion problem improves the effectiveness of the proposed STSR approach in dealing with spatial artifacts and preserves the spectral continuity of extracted objects.
3. The proposed STSR approach prevents spectral distortion over edge pixels by dividing an input image into the edge and non-edge pixels and eliminates spectral distortion over object classes and refinement of spectral and spatial responses of edge pixels in fused results.
4. A general optimization problem is exploited in the STSR approach to predict dictionary matrix along with sparse coefficients in a well-constraint sparse model.
5. The proposed approach is tested on real satellite data with a variety of object classes (OLI and MSI images from Tehran and Khorasan-e Razavi datasets), and experiments show that the proposed STSR approach can achieve reliable fusion results with lowest spectral distortion possible.

### Related work

Various spatiotemporal image fusion models have been proposed for different applications. Among them, some models are more typical and are widely used. These models require two coarse spatial resolution images captured at different times and one fine image as input to combine their merits. The most typical models include the spatial and temporal adaptive reflectance fusion model (STARFM^16^), unmixing-based data fusion (UBDF^17^), one-pair dictionary learning (OPDL^6^), Fit-FC^18^ (a three-step model consisting of regression model fitting, spatial filtering, and residual compensation), linear mixing growth model (LMGM^2^), and flexible spatiotemporal data fusion (FSDAF^19^), which are described briefly below.

STARFM^16^ assumes that the spectral difference between two different sensors is constant for homogeneous areas over time. A temporal weight factor is then deployed over a moving window to predict the behavior of the mixed land cover types using spectrally similar neighboring pixels. In this model, geometric misregistration, radiometric inconsistency between two sensors, and heterogeneity of the landscape cause drastic degradation of fused results. UBDF uses a linear spectral mixing model to predict the fine image by employing a constrained least square model with a moving window. In this model, fine pixels are assumed as pure end-members, and coarse pixels are linear combinations of the pure end-members. This assumption neglects the possibility of misregistration of geometric features and radiometric inconsistency over real satellite data. Thus, this model is recommended for geometrically and radiometrically corrected input images, and it is not ideal for real satellite data fusion. OPDL is a two-layer sparse representation (SR) framework that assumes sparse coefficients for coarse and fine images acquired from a specific scene are the same (which is not the case in real satellite data). In this model, temporal changes can be transferred using a high-pass modulation. Fit-FC^18^ establishes a linear regression model to capture temporal changes and apply them to fine pixels. Then, a weight function is defined to adjust the spectral response of a moving window based on nearest similar neighboring pixels, followed by interpolation of residuals over fine pixels. Although Fit-FC can deal with strong temporal changes, it fails to deal with high radiometric inconsistency due to disregarding the correlation between two coarse input images^7^.

LMGM is an unmixing-based fusion model that solves a linear system to estimate a constant growth rate for each end-member over time by selecting sufficient neighboring pixels via a moving window. Fusion results from this model are robust in dealing with the radiometric inconsistency yet fail to deal with geometric misregistration. FSDAF is an improved unmixing-based fusion model similar to LMGM except for considering the whole image instead of a moving window for selecting similar pixels to end-members. An interpolation step for residual compensation and smoothing the neighboring pixels is also conducted to achieve robust fusion results. FSDAF is recommended for spatiotemporal fusion applications based on the best performance among other proposed fusion models in the literature7. Results from the FSDAF model showed better preservation of spatial details and improvements in the accuracy of fused results compared to STARFM and UBDF20. Therefore, among the common spatiotemporal fusion approaches, Fit-FC and FSDAF are selected in this study to be compared with the proposed STSR approach because of their remarkable contribution in the spatiotemporal fusion of satellite data and the availability of source codes.

## Material and methods

### Sentinel-2 and landsat-8 data

The twin Sentinel-2 satellites are composed of Sentinel-2A and -2B satellites with a similar design that orbit the Earth in the same path with a 180° delay. Both platforms can revisit a particular location on Earth’s surface every 10 days (5 days when considering both satellites). The existence of clouds and shadows in a scene increases the time to capture a cloud-free Sentinel-2 image to more than five days for areas with a higher chance of covering with clouds (even several months). Sentinel-2 carries the MSI sensor with a 10 m GSD with 13 spectral bands on board. Vis/NIR spectral bands from MSI were selected and used as the input fine data in this study.

Spatiotemporal image fusion techniques require one pair of coarse- and fine-spatial resolution images captured simultaneously and one coarse spatial resolution image captured at a time different from the image pair. Real satellite data from OLI and MSI were selected as coarse and fine input images. Hereafter, we call the real coarse- and fine-spatial resolution images acquired at the same time as OLI_1_ and MSI_1_, respectively, and the other real coarse spatial resolution image as OLI_2_. As the reference fine spatial resolution image, the corresponding real MSI image captured at the same time as OLI_2_ is then used to assess the obtained results and is called MSI_2_.

### The proposed STSR approach

**Figure 1:**
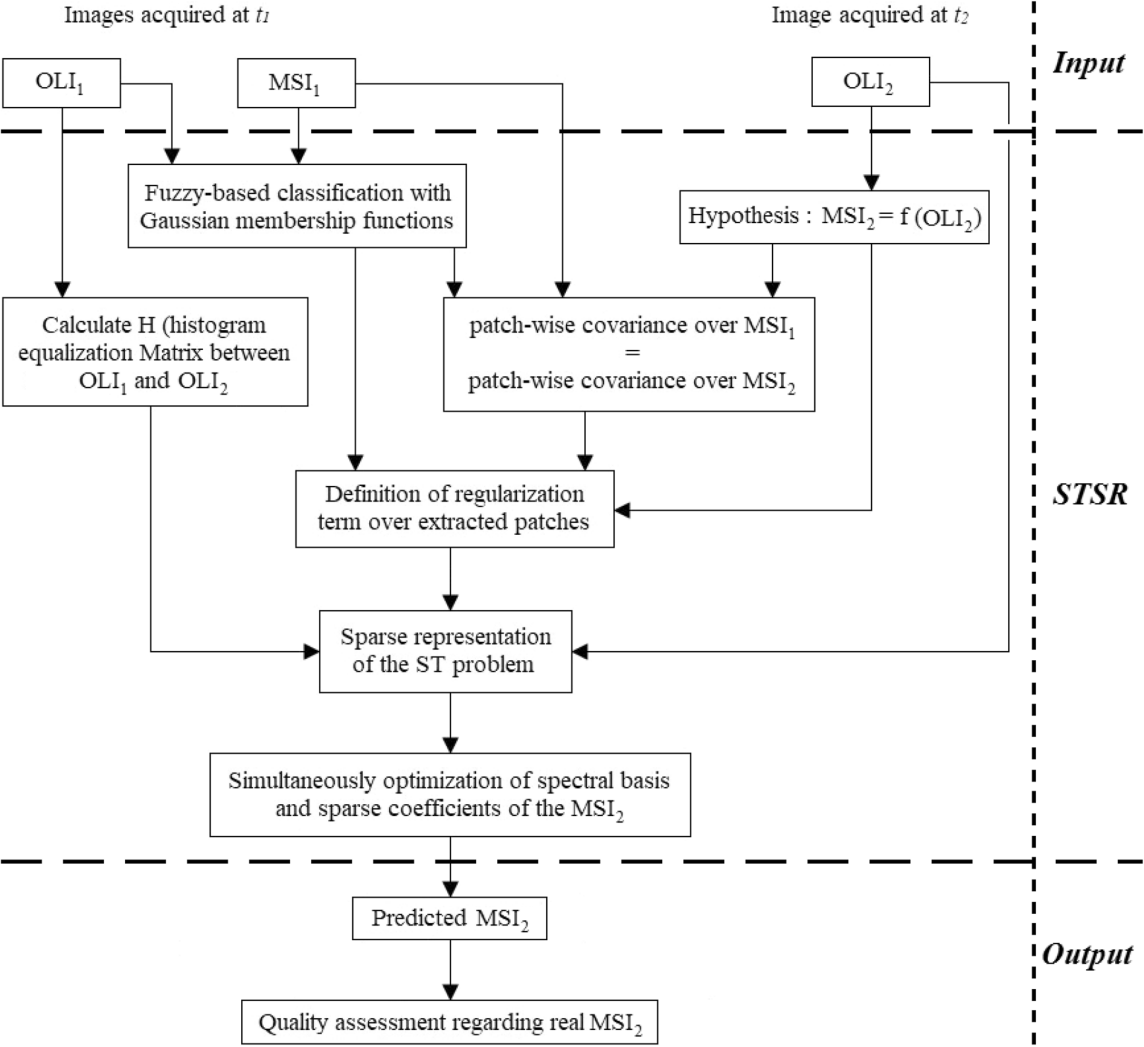
Flowchart of the proposed STSR approach.

This study introduces a new spatiotemporal fusion approach based on an SR model. The overall architecture of the proposed STSR approach is depicted in Fig. 1. The goal of this study is to predict an unavailable fine spatial resolution image using the corresponding coarse resolution image based on the spatiotemporal framework developed over another available coarse–fine pair image. One OLI_1_, one MSI_1_, and one OLI_2_ are considered inputs, and then, an image corresponding to MSI_2_ is predicted using the proposed STSR approach and compared to the real MSI_2_. According to Fig. 1, the proposed approach includes: (a) creating a fuzzy classification algorithm to improve the ability to distinguish different objects, especially over the object boundaries, where spectral mixing usually happens; (b) adopting membership functions in the fuzzy classification approach to identify the best pixels for edge candidates and improve their spectral response to achieve a more reliable fused result; (c) defining prior rules to adjust spatial covariance and equalize spectral response between input images and add them as constraints to the SR model; and finally (d) providing a well-constraint sparse model to estimate dictionary matrix along with sparse coefficients to predict fused results.

### Fuzzy classification algorithm

The fuzzy classification algorithm can deal with noisy data according to the characteristics of membership functions in converting crisp mathematical data to linguistic variables^20^. Local spectral variations, especially over the object boundaries—where the most spectral mixing occurs—reduce the quality of fusion results. Object-based procedure towards a fusion problem improves the effectiveness of the fusion approach in dealing with spatial artifacts and preserves the spectral continuity of extracted objects. Thus, an object-based classification method is required before sparse coding to extract objects, detect edge pixels, and prevent spectral mixing by assigning object classes with the most spectral similarity to edge pixels. Many researchers have suggested fuzzy inference systems (FISs) for such classification^20^. Firstly, the connected components of input MSI_1_ are extracted, followed by a merging step to eliminate the isolated components. Initial candidates for object boundaries are pixels with high spectral variances. The geometric pattern (GP) is derived from the morphology of the extracted components. Components with near- rectangular or circular shapes are assumed to be segments with high GP values. Therefore, to define components with higher values for GP, at least one of the following geometrical conditions need to be satisfied: (1) length-to-maximum width ratio more than 3/2, and an area close to length multiplied by maximum width, and (2) approximately equal distance from center to edge in four cardinal directions. The GP will be a matrix with the same size as MSI_1_ consisting of arrays of 1 for components with high GP and 0 for the rest of the components. Local variances over connected components (Var) are also extracted from MSI_1_. Secondly, the normalized difference vegetation index (NDVI) and the normalized difference water index (NDWI) are extracted from the input MSI_1_.

Then, an FIS approach is used to locate the edge pixels more accurately from extracted boundaries of segments by minimizing within-class variations. Fuzzy {if–then} rules are defined according to Table 1, based on Var, GP, NDVI, and NDWI. As shown in Table 1, three membership functions {low, moderate, and high} are considered for each input variable and output object class. A total of six object classes, namely, impervious surface, cropland, vegetated area, waterbody and wetland, bare soil, and others, are selected as output object classes. To use the fuzzy rules, first, the values of the variables are normalized within [0,1], with 1 and 0 corresponding to the highest and lowest possible values for each variable, respectively. Then, nonlinear Gaussian functions are selected for the membership functions. As an example, in Table 1, if Var and GP for a segment are {low} and {low}, respectively, then the impervious surface will be {low}. If the {low} membership values are activated for all object classes of a segment, it will be labeled as “Other.”

**Table 1:** Proposed fuzzy rules for the determination of each object class by having Var, GP, NDVI, and NWDI.

Object class	Membership functions		
	Low	Moderate	High
Impervious surface	Low Var + low GP	High Var + moderate GP	High Var + high GP
Cropland	High Var + low GP	Low Var + moderate GP	Low Var + high GP
Vegetated surface	Low NDVI + low Var	Moderate NDVI + high Var	High NDVI + high Var
Waterbody and wetland	Low NDWI	Moderate NDWI	High NDWI
Bare soil	Low NDWI + low NDVI + High Var	Low NDWI + low NDVI + Moderate Var	Low NDWI + low NDVI + low Var

After labeling all the segments in the input MSI_1_, membership functions of non-edge 8-nearest neighbors (with 10 m spatial resolution of MSI_1_) of the edge pixel are used to assign an accurate label to an edge pixel. The mean membership functions of the non-edge neighboring pixels (*MF*_*p*_) calculated from an inverse Euclidian distance determines the label of the edge pixel (Eq. 1)

1 $$MF_{P} = \sum\limits_{P} {\frac{{F_{P} }}{{\sqrt {\left( {x_{i} - x_{j} } \right)^{2} + \left( {y_{i} - y_{j} } \right)^{2} } }}}$$

where *P* is the number of non-edge neighboring pixels adjacent to an edge pixel, *F*_*p*_ is the memberships of the pixel for all object classes of non-edge neighboring pixels adjacent to the edge pixel, *d*_*i,j*_ is the Euclidean distance between the pixel located on edge (*x*_*i*_*,y*_*i*_) and its non-edge neighboring pixels (*x*_*j*_*,y*_*j*_). The label (object class) for each edge pixel (*C*_*e*_) is assigned based on the highest value for *MF*_*n*_ calculated from the non-edge 8-nearest neighboring pixels. This approach prevents spectral distortion over predicted edge pixels followed by a refinement of spectral and spatial responses and eliminates spectral distortion over predicted object classes.

### Sparse representation

SR is a technique that can decompose image signals to a linear combination of a few components from a predefined or learned dictionary, and infer those components (known as spectral bases), to extract salient features for increasing the capability of image reconstruction^21^. SR models tend to construct an over-complete dictionary over input images by dividing them into several overlapping patches to achieve meaningful representation of the source images^22^. SR models outperform many other image fusion methods’ capabilities, from stability and robustness to misregistration. These models define a trade-off between enhancing spatial detail and maintaining of spectral information to reduce spectral distortion^23^. Although both spatial and spectral information is considered simultaneously, defining an appropriate dictionary in SR models is necessary^24^.

Sparse coding represents a signal sparsely using an overcomplete dictionary and obtains promising performance in practical image processing applications, especially for signal restoration tasks. Equation (2) shows the most common form of sparse coding for multi-view problems in the form of an optimization problem15

2 $$\mathop {\min }\limits_{D,S} \sum\limits_{V} {\left\| {X^{T} - DS} \right\|^{2} + \beta \left\| S \right\|}$$

where *X* and *D*, are multi-view images with *V* spectral bands and the corresponding dictionary matrix, respectively, *S* is the sparsest possible representation matrix, known as the sparse matrix that satisfies Eq. (2), and *β*||*S*|| is a regularization function that improves the learning performance of dictionary matrix^25^. *β* is a constant value that controls the sparsity of the sparse matrix. The optimized value for *β* is estimated using an optimization method that maximizes the peak signal-to-noise ratio (PSNR) for all spectral bands of the predicted MSI_2_^26^. According to the literature, the feature sign search (*l*_1_ least squares) has been extensively used to optimize the sparse matrix, while the Lagrange dual method is used to optimize the dictionary matrix^27^. The optimization problem (Eq. 2) tries to minimize the difference between the product of *D* and *S,* and *X*, and estimate *X* more accurately. The optimization of SR parameters in remote sensing problems is severely ill-posed, with no unique solution. Thus, prior information is required to provide a unique solution for SR models.

### Implementation of the proposed approach

According to Fig. 1, for the prediction of the fine spatial resolution image, MSI_2_ is considered as a function of OLI_2_ image. Moreover, a good prediction of the unknown fine image requires a well-constraint SR model. Spatial and spectral relevance between OLI_1_ and MSI_1_, along with relationships between OLI_1_ and OLI_2_ are exploited as constraints to the SR model to predict the target MSI_2_.

A histogram equalization matrix is used to adjust spectral differences between input OLI images. Furthermore, FIS with nonlinear Gaussian membership functions is used to extract robust spatial and spectral constraints. It is then deployed over the MSI_1_ and upsampled OLI_1_ to assign labels to pixels with similar spectral characteristics. Results from the fuzzy classification are refined by a region growing and merging step for eliminating small objects. Then, the classified image is downsampled to the size of OLI_1_ followed by eliminating the isolated pixels. Investigation of the membership functions of fuzzy classification results for pixels located on the boundaries of adjacent objects reveals the best edge candidates. The label of an edge candidate in OLI_1_ is defined by the largest count of pixels of a specific label that occurred within the 3 × 3 corresponding pixel window in the MSI_1_.

Brightness shift or global spectral differences caused by the relative position of the sun and satellite sensor to the observation point and atmospheric condition between the two different acquisition times is preserved in the proposed approach by introducing a local histogram equalization constraint (*H*) in the SR model (Eq. 3). *H* was calculated separately for each spectral band of the OLI image. This constraint compares and matches corresponding local histograms over the objects in the input OLI_1_ and OLI_2_. Extracted objects from the fuzzy classification are used as local patches in the histogram equalization step to preserve the continuity of the spectral response in the fused result.

**Figure 2:**
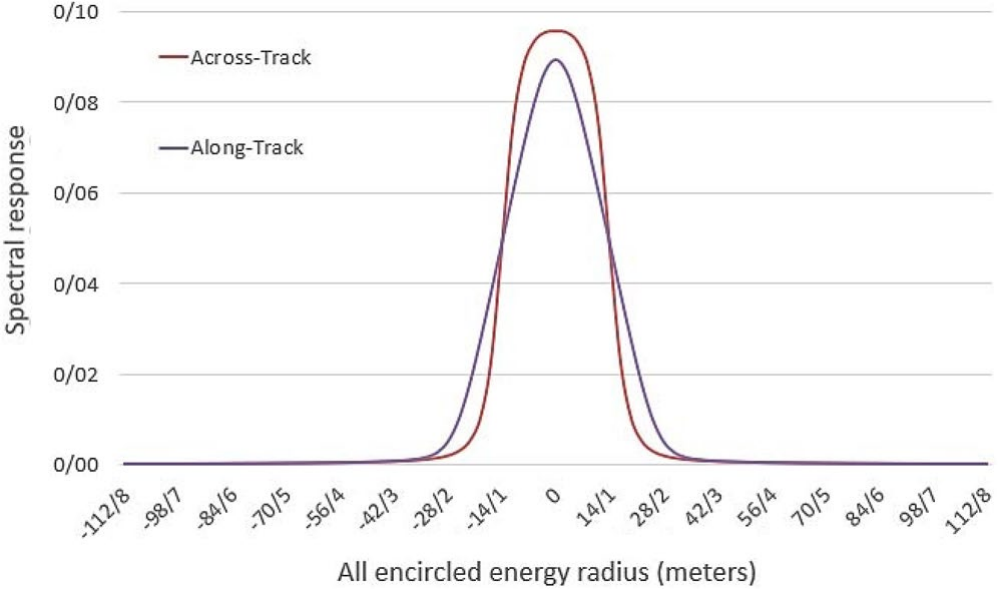
Across- and along-track spectral response of neighboring pixels of a given pixel to the energy acquired by the OLI B4 (red channel)^28^.

Furthermore, another constraint is defined in the proposed approach to extract the spatial information: covariance between the same objects of the MSI_1_ and MSI_2_ over a short period remains the same with slight changes that can be neglected. Thus, the covariance between OLI_1_ and MSI_1_ is equal to the covariance of those images captured at any other time (Eq. 3). Therefore, Eq. (3) is proposed to add spatial and spectral constraints into the SR model for predicting MSI_2_

3 $$\left\{ {\begin{array}{*{20}l} {{\text{HR - MSI - t}}_{i} :} \hfill & {X_{i} = D_{i} \times S_{i} } \hfill \\ {{\text{LR - MSI - t}}_{i} :} \hfill & {y_{i} = X_{i} \times R \otimes B \otimes H \to y_{i} = D_{i} \times S_{i} \times R \otimes B \otimes H} \hfill \\ {{\text{Cov}}(X_{i} ,Y_{i} ):} \hfill & {{\text{Cov}}(X_{1} ,Y_{1} ) = {\text{Cov}}(X_{2} ,Y_{2} )} \hfill \\ \end{array} } \right.$$

where *y*, *Y*, and *X* are the OLI, upsampled OLI that is resampled to the size of MSI (Eq. 3), and MSI, respectively. *i* denotes the acquisition time and can be set to 1 or 2. *X*_*i*_ is the target MSI_2_ when *i* = *2*. *H* implies the local histogram equalization matrix between OL_1_ and OLI_2_, over object classes in the input images. *D*_*i*_ and *S*_*i*_ are dictionary matrix and representation coefficients, respectively. They may vary depending on the acquisition time. *R* is the resampling matrix that downsamples input MSI to the size of OLI, and *B* is the proposed blurring matrix. Both *R* and *B* are physical constraints of the OLI sensor and are constant. *B* is defined according to the modulation transfer function (MTF) or point spread function (PSF) of the imaging system, whichever is available. The PSF is the essential characteristic of a sensor that defines the sensor’s spatial response to a point energy source within the instantaneous field of view. In this study, PSF is used to reconstruct *B* in Eq. (3). The spreadsheet of prelaunch measured PSF values for each spectral band of the OLI sensor in both the along- and across-track directions is provided by the US geological survey (USGS)^28^. According to this data, nearly 96.9% of all energy received by the OLI sensor encircles in a one-pixel radius of any given pixel for each spectral band. Similarly, a radius between 3 and 5 pixels of the MSI is suggested for the radius extension of the PSF^29^, equivalent to almost a one-pixel radius of the OLI sensor. Consequently, only 8-neighbors of a pixel in the OLI image are used to calculate the proposed blurring matrix. PSF for each spectral band was then simulated with a 3 × 3 Gaussian blurring matrix. The spectral response of neighboring pixels of a given pixel to the energy acquired by the OLI B4 (red channel) in both across- and along-track directions is shown in Fig. 2.

To calculate the covariance between input images for a specific time, OLI image (*y*) should be upsampled to the spatial size of MSI (*X*). Therefore Eq. (4) is proposed as below

4 $$Y_{i} = y_{i} \times R^{ - 1}$$

Cov(*X*_*i*_,*Y*_*i*_) indicates the proposed covariance constraint that is the covariance between *X*_*i*_ and *Y*_*i*_, and can be expressed as Eq. (5)

5 $${\text{Cov}}\left( {X_{i} ,Y_{i} } \right) = {\text{Cov}}\left( {D_{i} \times S_{i},\,\,\,D_{i} \times S_{i} \times R \otimes B \otimes H \times R^{ - 1} } \right)$$

Within-class covariance between the objects in *X*_*i*_, and corresponding objects in *Y*_*i*_, for both acquisition times, are calculated/predicted and subtracted implicitly to minimize the spectral variations due to different acquisition times and preserve continuity over objects. Therefore, we propose Eq. (6)

6 $$\mathop {\arg \min }\limits_{{D_{2} ,S_{2} }} \left\| {y_{2} - D_{2} \times S_{2} \times R \otimes B \otimes H} \right\|_{2} + \left\| {\sum\limits_{n = 1}^{N} {{\text{Cov}}\left( {X_{1}^{n} ,Y_{1}^{n} } \right) - {\text{Cov}}\left( {X_{2}^{n} ,Y_{2}^{n} } \right)} } \right\|_{2}$$

where *N* denotes the total number of labeled segments resulting from the fuzzy classification of *X*_*1*_. In practice, the dictionary matrix and sparse coefficients for the reference fine image (*D*_2_ and *S*_2_, respectively) are unknown with no unique solution. Therefore, we consider a regularization term to add object-based prior information to Eq. (6). According to this equation, the corresponding sparse matrix of MSI_2_ (*S*_2_) has to be minimized to achieve a unique solution. Spectral similarities between edge candidates and neighboring objects based on fuzzy membership functions are used to add edge details into the SR model. Therefore, the object-based spectral similarity parameter is proposed in this study to preserve spectral continuity over fused results (Eq. (7))

7 $$\mathop {\arg \min }\limits_{{D_{2} ,S_{2} }} \left\| {y_{2} - D_{2} \times S_{2} \times R \otimes B \otimes H} \right\|_{2} + \left\| {\sum\limits_{n = 1}^{N} {{\text{Cov}}\left( {X_{1}^{n} ,Y_{1}^{n} } \right) - {\text{Cov}}\left( {X_{2}^{n} ,Y_{2}^{n} } \right)} } \right\|_{2} + \varphi \left( {S_{2} } \right)$$

where *φ*(*S*_2_) is the proposed regularization term that provides additional prior information to the sparse problem, defined by Eq. (8)

8 $$\varphi \left( {S_{2} } \right) = \beta \left\| {S_{2} } \right\|_{1} + \alpha \sum\limits_{i = 1}^{N} {\left\| {D \times s_{{2_{j} }} - \sum\limits_{{j \in C_{N} }} {\omega_{i,j} D \times s_{{2_{i,j} }} } } \right\|}_{2}$$

where ||*S*_2_||_1_ is called the sparsity constraint and is the sum of absolute values of all elements of *S*_2_, and *α* and *β* are regularization parameters. Optimized values for *α* and *β* are estimated using an iterative evolutionary optimization that maximizes the PSNR for all spectral bands of the predicted MSI_2_. *ω*_*i,j*_ is the weight function and is the inverse number of pixels consisting of a similar object to the edge pixel. The main objective of Eq. (8) is to reduce the spectral difference between an edge pixel and the average of total pixels in a similar neighboring object extracted by fuzzy classification. This will alleviate inconsistencies in spectral responses from obtained results in dealing with real satellite data. After implementing Eq. (8), feature sign search (*l*_1_ least squares) and Lagrange dual method were used to optimize *S*_2_ and *D*_2_, respectively. For detailed information on the optimization method, readers are encouraged to refer to27.

### Performance evaluation method

An image fusion process might produce artifacts both spatially and spectrally, affecting the quality of the fusion products^30^. In this study, some popular image fusion quality metrics (IFQMs) were used to evaluate the performance of the spatiotemporal fusion results. In general, IFQMs are sorted into spatial and spectral categories. It is suggested to deploy spatial and spectral IFQMs in collaboration together to investigate the quality of fused results more accurately. Spectral IFQMs, including root-mean-square error (RMSE)^31^, relative dimensionless global error in synthesis (ERGAS)^32^, universal image quality index (UIQI)^33^, and spectral angle mapper (SAM)^34^, were used along with spatial IFQMs, including PSNR^35^39, and average gradient (AG)^36^, to evaluate the quality of the fusion results. Another IFQM that comprises both spectral and spatial aspects is the radiometric and geometric (RG) index (Eq. 9)^37^, which was introduced to evaluate the quality of fused results in a pansharpening procedure. RG index originally uses edge and background components from a fine spatial resolution panchromatic image and a multispectral coarse spatial resolution, respectively, to compare them with the edge and background components of fused result quantitatively. We generalized the RG index to evaluate the effectiveness of spatiotemporal fusion approaches by the replacement of input images with a real MSI_2_. Different quality indices can be used to compute both components for any fused results. SAM is selected as the quality index to calculate the RG index. Ideal values for the *SAM*_*RG index*_ are close to zero.

9 $$\begin{gathered} {\text{SAM}}_{Geometric} = \arccos \left( {\frac{{\left\langle {E,F} \right\rangle }}{{\left\| E \right\|_{2} .\left\| F \right\|_{2} }}} \right)\,\,\,\,\,\,\,\,\,\,\,\,\,\,\,\,\,\,\,\,(I) \hfill \\ {\text{SAM}}_{Radiometric} = \arccos \left( {\frac{{\left\langle {B,F} \right\rangle }}{{\left\| B \right\|_{2} .\left\| F \right\|_{2} }}} \right)\,\,\,\,\,\,\,\,\,\,\,\,\,\,\,\,\,\,\,\,(II) \hfill \\ (I),(II) \Rightarrow {\text{SAM}}_{RG\,index} = {\text{SAM}}_{Geometric} \times W_{Geometric} + {\text{SAM}}_{Radiometric} \times W_{Radiometric} \hfill \\ \end{gathered}$$

where *F* is the predicted MSI_2_ from a fusion approach, and *E* and *B* are edge and background components extracted from real MSI_2_, respectively. *W*_*geometric*_ and *W*_*radiometric*_ are weighting factors for geometric and radiometric components, respectively, and are calculated using the ratio between edge and background pixels and the total number of pixels in real MSI_2_. Among the common spatiotemporal fusion approaches, Fit-FC and FSDAF were implemented to quantify the performance of the proposed STSR approach and compare results from the proposed STSR approach. IFQMs usually evaluate the quality of fused pixels regardless of spatial, spectral, and textural behaviors. They do not report the damage caused by the fusion methods to the continuity of features and homogeneity of the objects. This shortcoming can be considered as a drawback to the IFQMs in the field of image fusion and can be addressed in future studies.

## Results and discussion

In order to evaluate the effectiveness of the proposed object-based STSR image fusion approach, predicted MSI_2_ from the STSR approach was compared to the results from other well-known and popular spatiotemporal image fusion methods. Spatial and spectral IFQMs were used to compare results and analyze the performance of the spatiotemporal fusion methods. The focus of this study is on the dataset, algorithm performance analysis, and discussion on the reasons for any possible anomalies in experimental results. All experiments were programmed using MATLAB R2018b software in a Microsoft Windows 10 environment on a desktop PC equipped with an Intel® Core™ i7-6700 K processor (8 M Cache, up to 4.20 GHz), and 12 GB of RAM.

### Dataset

**Figure 3:**
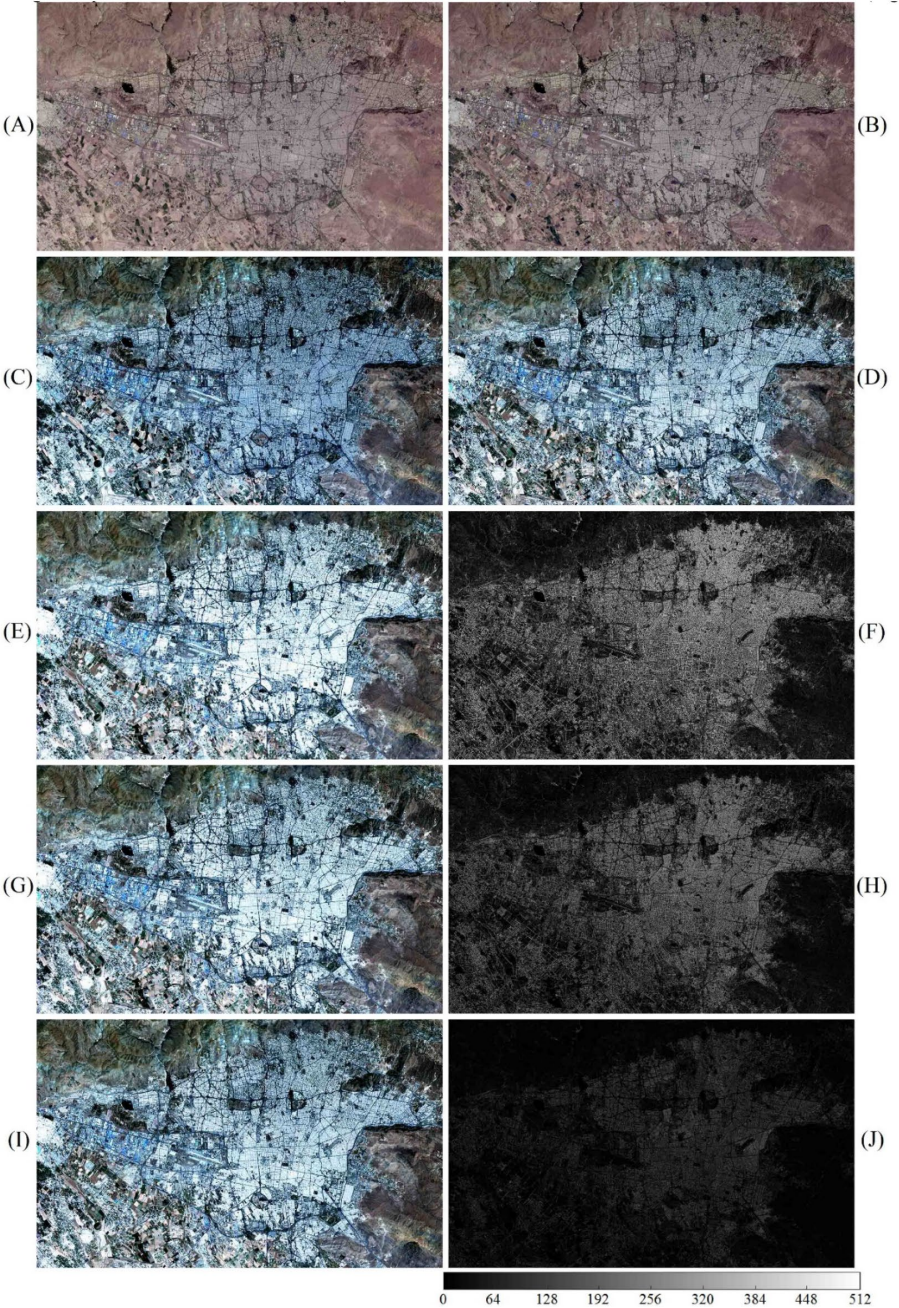
True color input images acquired from Tehran Province; (**A**, **B**) Upsampled OLI_1_ and OLI_2_, (**C**, **D**) corresponding MSI_1_ and MSI_2_, (**E**, **G**, **I**) predicted MSI_2_ using Fit-FC, FSDAF, and STSR approach, and (**F**, **H**, **J**) intensity difference map between predicted MSI_2_ and real MSI_2_. Scale bar shows the difference between predicted and real MSI_2_ on a 12-bit basis.

**Figure 4:**
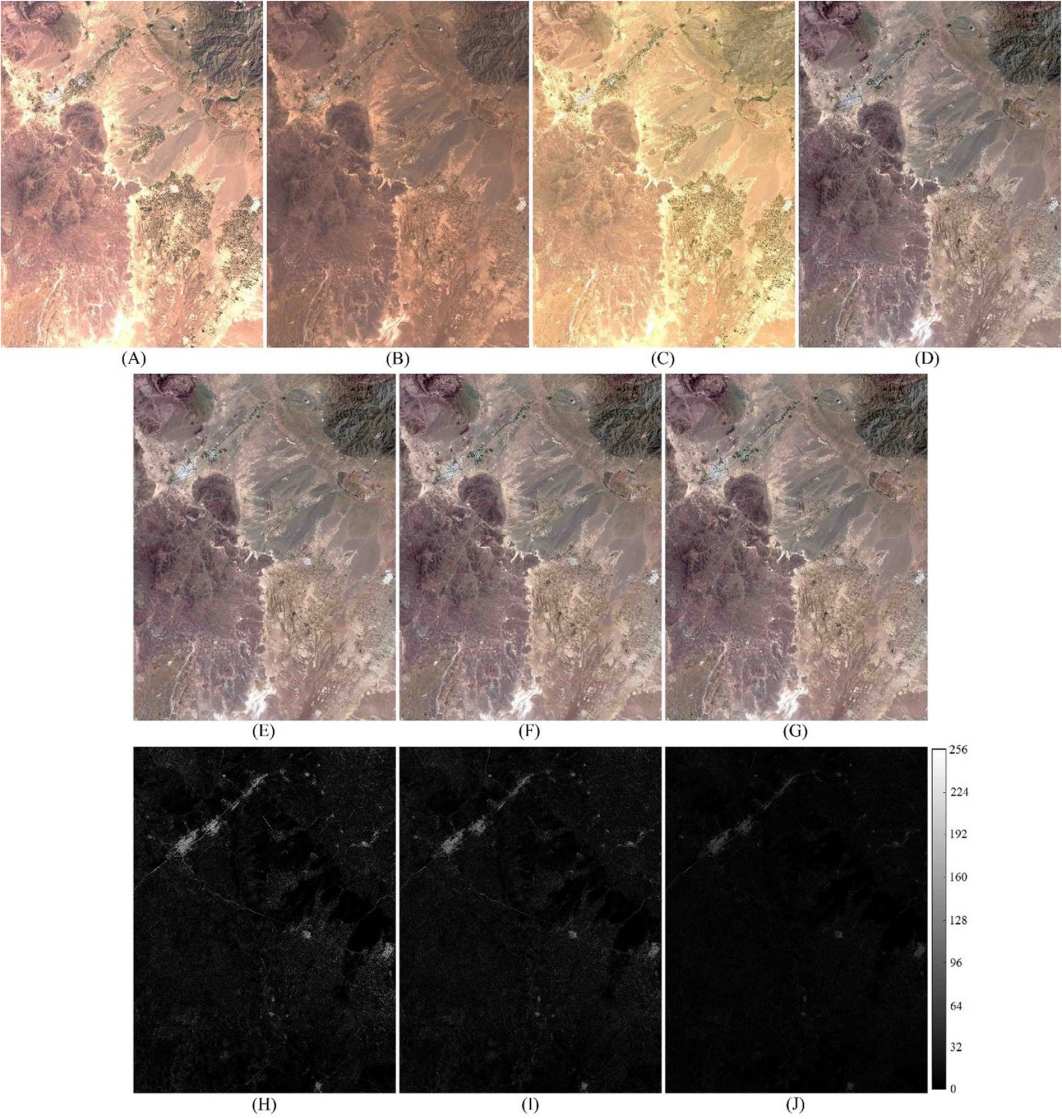
True color input images acquired from Khorasan-e Razavi Province; (**A**, **B**) upsampled OLI_1_ and OLI_2_, (**C**, **D**) corresponding MSI_1_ and MSI_2_, (**E**, **F**, **G**) predicted MSI_2_ using Fit-FC, FSDAF, and STSR approach, and (**H**, **I**, **J**) intensity difference map between predicted MSI_2_ and real MSI_2_. Scale bar shows the difference between predicted and real MSI_2_ on a 12-bit basis.

The proposed STSR image fusion approach requires two available OLI images acquired at two different times and an MSI acquired simultaneously with one of the available OLI images. The three input images are input to the STSR approach to predict an unknown MSI simultaneously acquired with the OLI_2_. We utilized real satellite images acquired over Tehran Province (35° 43′ N, 51° 24′ E) on June 29, 2019 and June 15, 2020 (Fig. 3A–C), and over Khorasan-e Razavi Province in Iran (36° 18′ N, 59° 36′ E) on July 17, 2018 and May 4, 2019 (Fig. 4A–C). Real satellite images from Khorasan-e Razavi Province were used in this study because they contain various types of land-use/land-cover (LULC). Real satellite images from Tehran Province were also used to include urban features with spectral mixing, especially over object boundaries in the fusion process. Both Khorasan-e Razavi and Tehran datasets contain four Vis/NIR spectral bands acquired from Landsat-8 OLI with a wavelength range of 0.45–0.88 µm, and 4 Vis/NIR spectral bands acquired from Sentinel-2 MSI with a wavelength range of 0.46–0.88 µm. The B2 (blue channel), B3 (green channel), B4 (red channel), and B8 (near-infrared channel) were selected from MSI as spectral bands of the fine image. Vis/NIR spectral bands from Landsat-8 OLI sensor with a 30 m GSD with a total of nine spectral bands were selected as the input coarse data. The B2 (blue channel), B3 (green channel), B4 (red channel), and B5 (near-infrared channel) were selected from OLI as spectral bands of the coarse image. Due to spectral similarity between vis/NIR spectral bands of Sentinel-2 MSI and Landsat-8 OLI sensors, a high spectral correlation exists between fine- and coarse-image that reduces spectral distortion in the fused result.

### Algorithm performance comparison

Various quality assessment indices were used to evaluate the effectiveness of the proposed fusion approach and compare its performance with well-known fusion approaches such as Fit-FC and FSDAF. Quantitative metrics for the spatiotemporal approach over different object classes in Khorasan-e Razavi and Tehran datasets are presented in Tables 2 and 3. Real MSI_2_ images (Figs. 3D and 4D) were used to assess the proposed STSR approach for spatial detail enhancement and spectral preservation. Fit-FC and FSDAF fusion approaches were also implemented on the two input datasets (Figs. 3E, G and 4E, F) to predict the MSI_2_, and compare their results with results from the proposed STSR approach. Predicted MSI_2_ from different spatiotemporal fusion approaches were compared to real MSI_2_ that was used as the reference fine image. Quantitative evaluation of spatiotemporal fusion approaches over different object classes in the Khorasan-e Razavi scene is presented in Table 2. Since the results from STSR, Fit-FC, and FSDAF approaches preserve the radiometric resolution (i.e., 12-bit) of the input images, reports for quantitative assessment of the fusion results are calculated based on the 12-bit radiometric resolution.

**Table 2:** Image fusion quantitative metrics for spatiotemporal problem for 12-bit MSI and OLI at two different acquisition times over different object classes for the Khorasan-e Razavi dataset.

Object class	Approach	RMSE	ERGAS	SAM	UIQI	PSNR	AG	SAM_*RG index*_		
								G	R	RG
Impervious surface	Fit-FC	39.095	1.599	18.73	0.9203	208.16	149.41	5.69	9.82	7.91
	FSDAF	36.948	1.512	16.22	0.9717	208.65	**192.98**	4.07	11.23	7.44
	STSR	**23.593**	**0.965**	**9.25**	**0.9924**	**212.54**	183.32	**1.91**	**7.05**	**3.56**
Farmland	Fit-FC	8.653	0.461	7.01	0.9840	221.26	111.57	2.99	5.30	4.07
	FSDAF	9.496	0.506	10.59	0.9965	220.45	**137.09**	3.43	6.47	4.85
	STSR	**8.221**	**0.438**	**3.18**	**0.9983**	**221.70**	125.79	**1.18**	**1.60**	**1.49**
Vegetated surface	Fit-FC	17.253	0.743	6.58	0.9900	215.26	97.35	2.39	5.16	3.98
	FSDAF	18.692	0.805	7.09	0.9976	214.57	**118.03**	2.54	5.72	4.13
	STSR	**16.710**	**0.720**	**4.43**	**0.9986**	**215.54**	117.39	**2.29**	**3.11**	**2.72**
Water body and wetland	Fit-FC	**2.803**	**0.101**	1.63	0.9951	**231.05**	90.47	0.70	1.56	1.01
	FSDAF	2.971	0.107	1.78	0.9986	230.54	97.88	0.74	1.62	1.24
	STSR	2.836	0.102	**1.02**	**0.9989**	230.96	**98.22**	**0.55**	**0.89**	**0.83**
Overall	Fit-FC	16.951	0.726	8.49	0.9723	218.93	112.20	2.94	5.46	4.24
	FSDAF	17.027	0.733	8.92	0.9911	218.55	**136.49**	2.70	6.26	4.42
	STSR	**12.840**	**0.556**	**4.47**	**0.9971**	**220.19**	131.18	**1.48**	**3.16**	**2.15**

Significant values are in bold.

**Table 3:** Image fusion quantitative metrics for spatiotemporal problem for 12-bit MSI and OLI at two different acquisition times over different object classes for the Tehran dataset. No object class with the farmland label was detected in this area.

Object class	Approach	RMSE	ERGAS	SAM	UIQI	PSNR	AG	SAM_*RG index*_		
								G	R	RG
Impervious surface	Fit-FC	92.619	2.601	25.96	0.8544	200.66	167.31	3.35	20.64	12.29
	FSDAF	81.236	2.159	21.45	0.9539	201.81	**244.06**	2.77	17.19	10.53
	STSR	**30.492**	**1.186**	**14.37**	**0.9896**	**210.32**	208.25	**1.88**	**7.38**	**3.71**
Farmland	Fit-FC	–	–	–	–	–	–	–	–	–
	FSDAF	–	–	–	–	–	–	–	–	–
	STSR	–	–	–	–	–	–	–	–	–
Vegetated surface	Fit-FC	19.568	0.307	5.36	0.9661	214.17	153.29	3.63	4.39	4.01
	FSDAF	23.755	0.372	9.23	0.9913	212.48	**170.98**	4.52	8.07	4.84
	STSR	**12.776**	**0.200**	**2.95**	**0.9983**	**217.87**	162.73	**2.03**	**3.10**	**2.59**
Water body and wetland	Fit-FC	0.909	0.104	1.97	0.9904	240.83	92.95	0.74	1.68	1.27
	FSDAF	0.987	0.113	2.65	0.9972	240.11	**119.06**	1.52	2.23	1.95
	STSR	**0.813**	**0.093**	**1.44**	**0.9990**	**241.80**	130.14	**0.49**	**0.91**	**0.82**
Overall	Fit-FC	37.699	1.004	11.10	0.9370	218.55	137.85	2.57	8.90	5.86
	FSDAF	35.326	0.881	11.11	0.9808	218.13	**178.03**	2.94	9.16	5.77
	STSR	**14.694**	**0.493**	**6.25**	**0.9956**	**223.33**	167.04	**1.47**	**3.80**	**2.37**

Significant values are in bold.

According to Tables 2 and 3, although FSDAF shows better RMSE values than Fit-FC over impervious surfaces, RMSE for Fit-FC is higher over more heterogeneous regions. Thus, FSDAF is more robust to spectral variations compared to Fit-FC. As shown in Table 2, RMSE values for Fit-FC over the waterbody and wetland object class show better spectral preservation compared to STSR. However, STSR outperforms Fit-FC for other object classes (Tables 2 and 3). Moreover, other IFQMs show a better performance for the proposed STSR approach over the waterbody and wetland object class. The reason for such false alarm in Fit-FC is the smoothed results due to the deployment of a local linear regression model in the modeling process. Since Fit-FC uses a local linear regression model to capture the temporal changes and deploys spatial filtering to smooth the results, some spatial details are lost in the modeling process. That explains the degraded results for Fit-FC according to UIQI and AG in Tables 2 and 3. UIQI measures the amount of data being transferred from reference fine image to fused image by calculating an average of spectral and contrast similarity between the images. Besides, spectral gradients decrease over smoothed predictions which affect the AG index. AG shows image intelligibility by representing the sum of horizontal and vertical spectral variations. Higher values show a better understanding of the image. However, spectral distortion over edge pixels increases the values for AG and shows better results with higher interpretability which is not true. That explains the higher values for AG over predicted results using FSDAF. PSNR is another IFQM that represents the power of distorting noise and is adjusted based on the radiometric resolution of the imaging system, which is equal to 12-bits in this study. A comparison between PSNR values shows promising results for all fusion methods. However, compared to other fusion approaches, STSR retrieves slightly more signals with less noise over impervious surfaces. In other words, the proposed STSR approach provides better spatial details over impervious and more complex regions compared to other methods. This is due to the object-based procedure towards the spatiotemporal fusion problem and the excessive number of objects in the Tehran dataset (Table 3 and Fig. 3).

**Figure 5:**
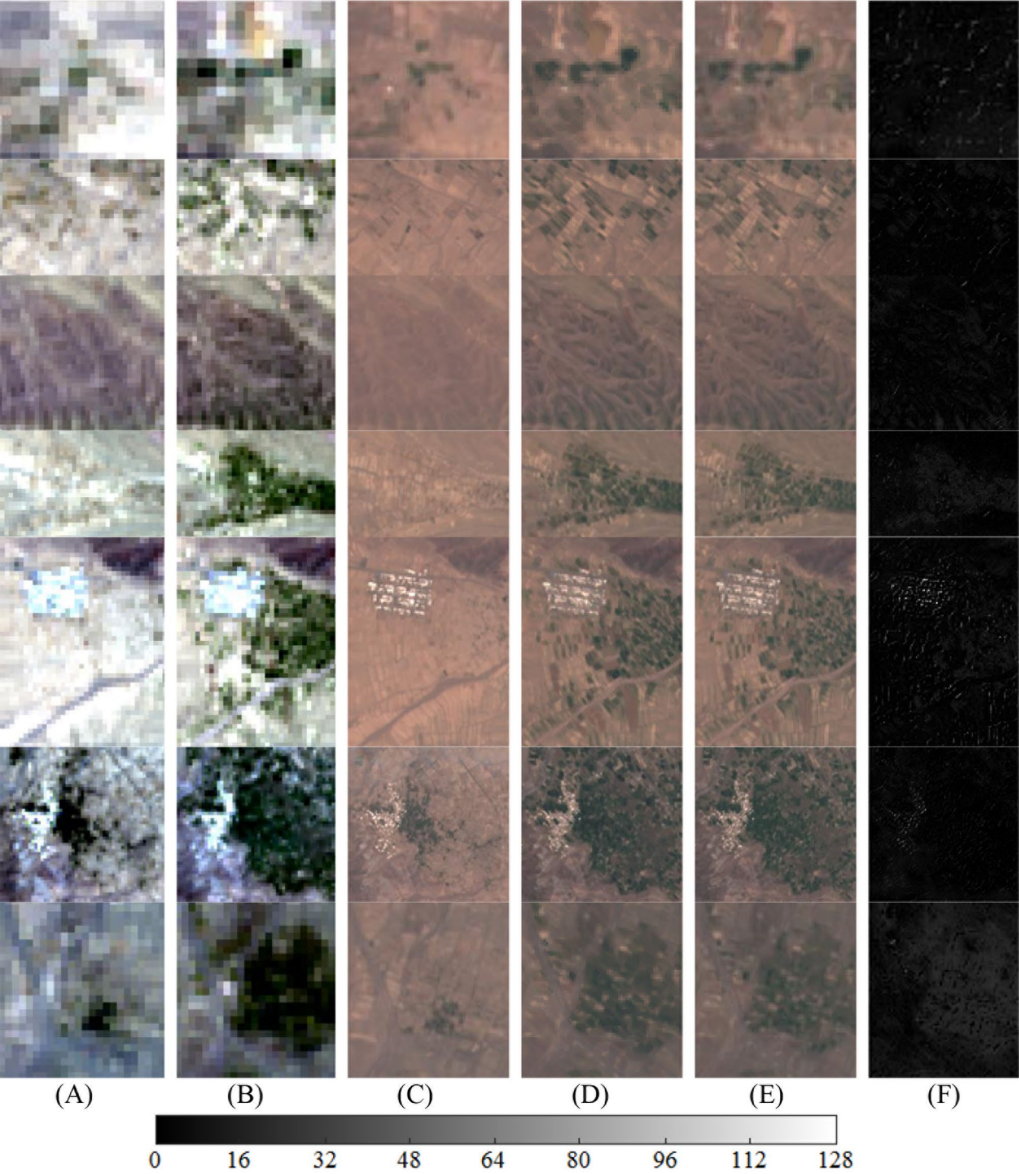
(**A**, **B**) True color OLI_1_ and OLI_2_, (**C**) true color MSI_1_, (**D**) predicted results from the proposed STSR approach, (**E**) true color real MSI_2_, (**F**) intensity difference map between predicted MSI_2_ and real MSI_2_. Scale bar shows the difference between predicted and real MSI_2_ on a 12-bit basis.

Based on the results from Tables 2, 3, Figs. 3, 4, and 5.

Figure 5, the proposed STSR approach exerted better performance over smooth regions than impervious and more complex regions, which is similar to the performance of other spatiotemporal methods. STSR preserves spectral information better than spatial information, which is a reason for temporal changes of the objects in the scene between the two acquisition times. According to the intensity difference map (Fig. 5F), the spectral similarity of predicted and real MSI_2_ images (Fig. 5D, E).

Figure 5 is a result of histogram equalization between the extracted labeled segments from the fuzzy classification in input images, which was introduced in the SR model as a constraint. The spectral continuity over the extracted segments from the fuzzy classification algorithm is another advantage of the proposed STSR approach. Minimizing the spectral variations over each object by introducing a patch-wise covariance parameter as a spatial constraint eliminates the brightness shift. Therefore, the proposed STSR approach smooths extreme differences between spectral responses at different acquisition times, eliminating noise pixels or pixels with a remarkable spectral change in a patch due to temporal change to reduce spectral distortions resulting from changes that are not captured in OLI image.

According to Fig. 5F, spectral distortions in the fusion process mainly occur over edge pixels where objects with different spectral responses are adjacent. Spectral mixing is a regular phenomenon over object boundaries, especially in rural and urban areas with high variation in object classes. Thus, assigning the exact object class to each edge pixel extracted from the fuzzy classification and approximating it with the similar object class in the SR model reduces the spectral distortion in the fusion results. Considering PSF is another way to predict an MSI from an OLI in the sense of spectral mixing. Spectral mixing of neighboring pixels caused by spatial degradation of data from MSI to OLI was modeled by a Gaussian blurring matrix, then added into the SR model to predict the unknown MSI_2_. It is used to create column D from column B in Fig. 5.

Urban areas contain more impervious objects, and therefore, more spectral variations are expected due to high spectral variations over impervious surfaces caused by the relative position of the sun and satellite sensor to the observation point. Therefore, higher spectral degradation is more likely over urban and complex areas than areas with fewer spectral variations, such as Khorasan-e Razavi dataset. By investigating spectral quality metrics, e.g., RMSE, ERGAS, SAM, and UIQI in Table 2, it can be seen that IFQMs for all spatiotemporal fusion methods are closer to their ideal values compared to those in Table 3. Better spectral detail preservation over smoother areas in the Khorasan-e Razavi dataset was expected due to fewer impervious objects than the Tehran dataset. However, Table 3 shows that an acceptable performance can also be obtained using the proposed STSR fusion approach on the Tehran dataset. The proposed STSR approach eliminates spectral distortion by assigning the best object class to each edge candidate, and reducing spectral differences among them.

STSR presents spectral characteristics of OLI_2_ and spatial characteristics of MSI_1_. It prevents spectral distortion over edge pixels and eliminates spectral artifacts while preserving spectral continuity over the extracted objects and hence, alleviating the common problem of detail loss. On the one hand, the proposed STSR approach preserves local changes that occurred at the time of acquisition of OLI_2_ and applies it to available MSI_1_ to predict the unknown MSI_2_ considering constant covariance between images at the two acquisition times. On the other hand, the object-based approach reduces errors caused by the misregistration of corresponding images acquired at different times.

According to the intensity difference maps (Figs. 3J, 4, 5J,F), the proposed STSR image fusion approach can efficiently deal with temporal changes. The maximum intensity difference between predicted MSI_2_ and real MSI_2_ from STSR is slightly more than 3.1% and 6.2% over regions with smooth and high spectral variations, respectively. Therefore, the proposed object-based STSR image fusion approach preserves almost 96.9% and 93.8% (vs. 92.4 and 80.7 for Fit-FC, 93.1 and 87.5 for FSDAF), of the spectral detail of the MSI image over smooth and urban areas, respectively.

According to results from our proposed STSR image fusion approach, we achieved the goals we set out at the beginning of this study. We proposed a fuzzy classification algorithm based on membership functions to extract different object classes and improve spectral responses over edge pixels to prevent unrealistic predicted results. Then, an object-based adjustment of spatial covariances and equalization of spectral responses were made on extracted objects to reduce spectral distortion in predicted results. A general well-constraint spatiotemporal SR model was then proposed over the real input satellite data to predict fused results. Finally, results from our proposed approach were compared to Fit-FC and FSDAF methods that show an outstanding improvement of the proposed STSR approach over real satellite images compared to other methods.

## Conclusions

In this study, a spatiotemporal image fusion approach was proposed to predict an unknown fine spatial resolution image from input real satellite data. When global fine spatial resolution images are not available, this approach can be used to predict dense fine spatial resolution images. For this, a set of real OLI images acquired at different times and an MSI acquired at the same time with one of the OLI images are selected for the proposed STSR fusion approach to predict the target MSI image corresponding to the other OLI image. A rule-based fuzzy classification algorithm was proposed to improve the ability to distinguish different objects in a scene and identify edge candidates more accurately. Results from the proposed STSR approach were compared to those of the Fit-FC and FSDAF as reliable methods available in the literature.

The findings of this study showed that the object-based procedure towards the spatiotemporal fusion problem improves the performance of the proposed STSR approach in dealing with spatial artifacts and preserves the spectral continuity of extracted objects. Therefore, the proposed STSR focuses on enhancing spectral responses of predicted edge pixels to prevent spectral distortion in fused results. An SR model was designed and implemented on real input satellite data to create the proposed STSR fusion approach. An object-based estimation of physical constraints and brightness shift between real input data was added to the SR model to create a unified framework to deal with real satellite data. Due to the underdetermined nature of the spatiotemporal fusion problem for remote sensing applications, a well-constraint SR model with the sparsest solution is necessary. Thus, different object-based prior information was added to the SR model to provide sufficient constraints to use as regularization parameters to solve the SR model. The proposed object-based STSR approach fuses spectral information regarding their spatial relationship and focuses on the enhancing of spectral information over edge candidates at the predicted result. Consequently, spectral distortion and discontinuity over continuous objects are reduced in predicted results. According to the results, a quantitative evaluation of the STSR approach obtained convincing values for both spatial and spectral quality metrics. Results from the quality assessment step show a promising performance of the proposed STSR approach considering spectral differences over objects between predicted and real fine satellite images. Evaluation of results from both study areas indicates that the proposed STSR approach can handle slight changes over objects made due to time. Thus, the proposed STSR approach is appropriate for dynamic monitoring of high-frequency phenomena over various types of LULCs.

## Data availability

Vis/NIR spectral bands of both OLI and MSI sensors are freely available to users and have global coverage. Sentinel-2 MSI data are accessible via the Copernicus open access hub^38^ server provided by the European space agency (ESA), and Landsat-8 OLI data is downloaded from the USGS Earth Explorer^39^ web portal. The datasets used in this paper are hyperlinked and available online at Tehran and Khorasan-e Razavi, respectively. Due to the launch of the twin satellite Landsat 9 on September 27, 2021, with a similar design to the Landsat-8^40^, the revisit time of the captured imagery will soon reduce in half with the twin satellites together. Radiometric precision of L9 has improved to 14-bit quantization resolution compared to 12-bit for L8. However, bandwidths and central wavelengths for all OLI spectral bands remained the same. Fusing L8-OLI and Landsat-9 OLI-2 (L9-OLI) images with Sentinel-2 MSI images to achieve a continuous time series of effective Sentinel-2 observations over the regions with a high probability of snow and cloud contamination will be a good opportunity for future studies. In future researches, we will focus on the spatiotemporal fusion of the twin Landsat OLI images with the twin Sentinel-2 MSI images to fill existing data gaps in the time series of fine images.
